# A Transcriptome Map of *Actinobacillus pleuropneumoniae* at Single-Nucleotide Resolution Using Deep RNA-Seq

**DOI:** 10.1371/journal.pone.0152363

**Published:** 2016-03-28

**Authors:** Zhipeng Su, Jiawen Zhu, Zhuofei Xu, Ran Xiao, Rui Zhou, Lu Li, Huanchun Chen

**Affiliations:** 1 State Key Laboratory of Agricultural Microbiology, College of Veterinary Medicine, Huazhong Agricultural University, Wuhan 430070, China; 2 Cooperative Innovation Center of Sustainable Pig Production, Wuhan 430070, China; University of Georgia, UNITED STATES

## Abstract

*Actinobacillus pleuropneumoniae* is the pathogen of porcine contagious pleuropneumoniae, a highly contagious respiratory disease of swine. Although the genome of *A*. *pleuropneumoniae* was sequenced several years ago, limited information is available on the genome-wide transcriptional analysis to accurately annotate the gene structures and regulatory elements. High-throughput RNA sequencing (RNA-seq) has been applied to study the transcriptional landscape of bacteria, which can efficiently and accurately identify gene expression regions and unknown transcriptional units, especially small non-coding RNAs (sRNAs), UTRs and regulatory regions. The aim of this study is to comprehensively analyze the transcriptome of *A*. *pleuropneumoniae* by RNA-seq in order to improve the existing genome annotation and promote our understanding of *A*. *pleuropneumoniae* gene structures and RNA-based regulation. In this study, we utilized RNA-seq to construct a single nucleotide resolution transcriptome map of *A*. *pleuropneumoniae*. More than 3.8 million high-quality reads (average length ~90 bp) from a cDNA library were generated and aligned to the reference genome. We identified 32 open reading frames encoding novel proteins that were mis-annotated in the previous genome annotations. The start sites for 35 genes based on the current genome annotation were corrected. Furthermore, 51 sRNAs in the *A*. *pleuropneumoniae* genome were discovered, of which 40 sRNAs were never reported in previous studies. The transcriptome map also enabled visualization of 5'- and 3'-UTR regions, in which contained 11 sRNAs. In addition, 351 operons covering 1230 genes throughout the whole genome were identified. The RNA-Seq based transcriptome map validated annotated genes and corrected annotations of open reading frames in the genome, and led to the identification of many functional elements (e.g. regions encoding novel proteins, non-coding sRNAs and operon structures). The transcriptional units described in this study provide a foundation for future studies concerning the gene functions and the transcriptional regulatory architectures of this pathogen.

## Introduction

During the past decade, a large number of bacterial complete genomes have been finished, providing the sequence basis for transcriptomics and proteomics. The genome sequence contains all the information of the functional elements including genes, non-coding RNAs, and UTRs, which are required for a system-level understanding of the survival mechanisms and pathogenesis of microorganism [1]. However, due to the inherent limitations of the computational methods for prokaryotic gene annotation, most of the present genome sequences are still at the preliminary stage to define accurate gene boundaries, to predict gene functions and regulatory elements, especially small non-coding RNAs (sRNAs) and UTRs. Therefore, to further take advantage of the genome sequences, the original genome annotations need to be revised for accurate identification and demarcation of all the functional elements in a genome.

Transcriptome of bacteria represents the entire transcripts expressed in a certain condition. Genome-wide transcriptome analysis enables researchers to improve the genome structural annotation and reveal the functional and regulatory architecture of the genome [2]. Microarrays have been widely applied to study gene expression patterns. The recently developed RNA sequencing (RNA-seq) is another important technique for transcriptome profiling, as several advantages compared with microarrays, such as low background noise, high throughput and high resolution[3–5]. Several recent studies have proven that RNA-Seq can comprehensively re-annotate the bacteria genomes and is useful for understanding the complexity of bacterial transcriptome and identifying previously unknown functional elements [6–8]. For example, RNA–Seq based analysis of the transcriptome of the *S*. *Typhi* has identified 40 novel non-coding RNAs and re-annotated a number of hypothetical genes [9]. Thirty-eight novel genes, 94 sRNAs and 278 operon structures in the *H*. *somni* genome have been characterized by using a transcriptional map generated by RNA-Seq[10].

*Actinobacillus pleuropneumoniae*, a Gram-negative pathogen of the *Pasteurellaceae* family, is the primary etiologic agent of porcine contagious pleuropneumonia, a severe respiratory disease causing great economic losses to the worldwide swine industry [11]. The genome of *A*. *pleuropneumoniae* JL03 was sequenced 7 years ago [12]. After that, a comparative genomic analysis has been done based on the 12 genomes of serovar reference strains, depicting genomic features associated with serovar diversity [13]. These *A*. *pleuropneumoniae* genome annotations were made by computational analysis based on gene prediction algorithms [12]. Thus, the existing annotations of the *A*. *pleuropneumoniae* genomes are far from complete. The precise gene structures, transcription units, expressed genes and sRNAs are required to be identified to explore more gene functions and regulatory modes to study *A*. *pleuropneumoniae* survival and infection mechanisms. Therefore, in this study, we used deep RNA-seq technology to characterize transcriptome structure of *A*. *pleuropneumoniae*. The single nucleotide resolution map generated helped to not only correct the previous annotation errors, but also identify novel protein coding regions, operon structures as well as sRNAs.

## Materials and Methods

### Bacterial strain and culture conditions

The bacterial strain used in this study is *Actinobacillus pleuropneumoniae* JL03 (a clinical isolate of serovar 3 [12]). It was grown at 37°C in tryptic soy broth (TSB) or on tryptic soy agar (TSA) supplemented with 10% (v/v) filtered cattle serum and 10 μg/ml nicotinamide adenine dinucleotide (NAD).

### RNA isolation, library construction and sequencing

In preparation for RNA isolation, *A*. *pleuropneumoniae* JL03 was cultured with rotation at 180 rpm to mid-log phase (optical density at 600 nm = 1.0). Total RNA was extracted using RNA Midiprep kit (Promega, Madison, USA) according to the manufacturer’s protocol. Total RNA was treated with RNase-free DNase (Invitrogen, Carlsbad, CA) to remove traces of genomic DNA. By using Agilient Bioanalyzer 2100 (Agilent Technologies, Santa Clara, CA), we checked the RNA integrity number (RIN) of total RNA to be greater than 8. Bacterial 16S and 23S ribosomal RNAs were removed with a MICROBExpress ^™^ kit (Ambion, TX, USA). Small RNAs (i.e., tRNA and 5S rRNA) were not removed with this enrichment step. The enriched mRNA was processed to a RNA-Seq library using the mRNA-Seq sample preparation kit (Illumina, San Diego, CA) following the manufacturer’s instructions. The constructed sequencing library was sequenced using the Illumina HiSeq 2000 platform (Illumina, San Diego, CA) at the Shenzhen Genome Institute (BGI, Shenzhen, China).

### RNA reads mapping and visualization

Before mapping of reads to the genome, the raw data were analyzed using FASTQC tool [14] (S1 Fig) and pre-processed to obtain high quality reads. FastQC reports revealed that our samples were successfully sequenced. Raw reads were quality-filtered using a reads trimming tool Trimmomatic with default settings [15]. We checked and cleaned all Illumina raw reads by removing the following types of sequences: those corresponding to adapters, those with low read quality (more than 40% of the reads with a base quality value lower than 20), and those containing more than 10% ‘‘N” bases. All clean reads were mapped to the *A*. *pleuropneumoniae* JL03 genome sequence (GenBank Accession number. CP000687) using the Burrows-Wheeler alignment tool (BWA) [16], with up to two base mismatches allowed. The reads that mapped to more than one location were discarded [17]. Only uniquely mapped reads were used for further analysis. The resulting data was transformed into SAM/BAM format with Samtools [18] to generate alignment results in a pileup format, which provided the signal map file and contained the count of reads per base. In-house Perl scripts were written to calculate the background expression. The sequence coverage per base was plotted and visualized using the genome browser Artemis [19].

#### Analysis of expressed intergenic regions of *A*.*pleuropneumoniae* JL03 genome

Prior to the analysis of expressed regions in the genome, we calculated the background expression with two criteria as previously reported [10, 20]. In brief, one criterion is that at least half of the intergenic region is assumed as not expressed; the other one criterion is that the coverage depth (reads per base) of expressed reads should be greater than the lower tenth percentile of all reads. Identification of UTR regions was performed by searching for the point of rapid coverage reduction (drop down to 1/10 of the mean coverage value of the gene) in the regions upstream of the start codon and downstream of the stop codon. Expressed intergenic regions (EIRs) within predicted operons [21] are difficult to determine the UTR regions and can be mis-classified as sRNAs. Hence these regions were discarded to minimize the number of false positives. To identify novel protein coding regions and correct start codon locations, sequences of EIRs were searched with BLASTX against sequences in NCBI non-redundant (nr) protein database followed by manual analysis and interpretation. If protein coding region was found in the EIRs, the ORF search was performed using the web program ORF Finder [22]. If no protein coding regions were found and a promoter or a Rho-independent terminator was present, the EIRs were classified as sRNA [10]. The Prokaryotic Promoter Prediction (PPP) program [23] and Transterm HP [24] were used to predict promoters and Rho-independent terminators. For functional annotation, all identified sRNA sequences were aligned with the sequences in the Rfam database [25]. To study the conservation of identified sRNAs among others genomes, the sRNA sequences were searched with blastn against the non-redundant, nucleotide database at NCBI.

#### Analysis of expressed annotated regions of *A*.*pleuropneumoniae* JL03 genome

Expressed reads with coverage above background were mapped onto the annotated genes of *A*. *pleuropneumoniae* JL03. Using the annotation information (gene loci) of *A*. *pleuropneumoniae* JL03 and the pileup file, the genes that had a significantly higher proportion of their length (at least 60%) covered by expressed reads were considered as to be expressed. Annotated regions below 60% coverage were considered as “not expressed” under the current experimental conditions. Similar measures have been used in other transcriptome profiling studies [26, 27]. The functions of genes expressed in the present study were classified according to the Clusters of Orthologous Groups (COG) databases [28]. For the identification of operons, we used the following rules according to previous studies [10, 29, 30]: (a) All the genes in an operon are expressed; (b) Genes in an operon are transcribed in the same orientation; (c) The intergenic regions between the genes in the operon are expressed. Operon structures identified in this study were compared with the computationally-predicted operon structures described by the Database for prokaryotic OpeRons (DOOR) [21] for verification and comparison.

### RNA-Seq data accession number

The RNA-Seq data used in this work have been submitted to the NCBI GEO (Gene Expression Omnibus) database with the accession number GSE70153. (For reviewer link: http://www.ncbi.nlm.nih.gov/geo/query/acc.cgi?token=itctauugrzqtxkp&acc=GSE70153)

## Results

### Reads mapping to the *A*. *pleuropneumoniae* JL03 genome

**Fig 1:**
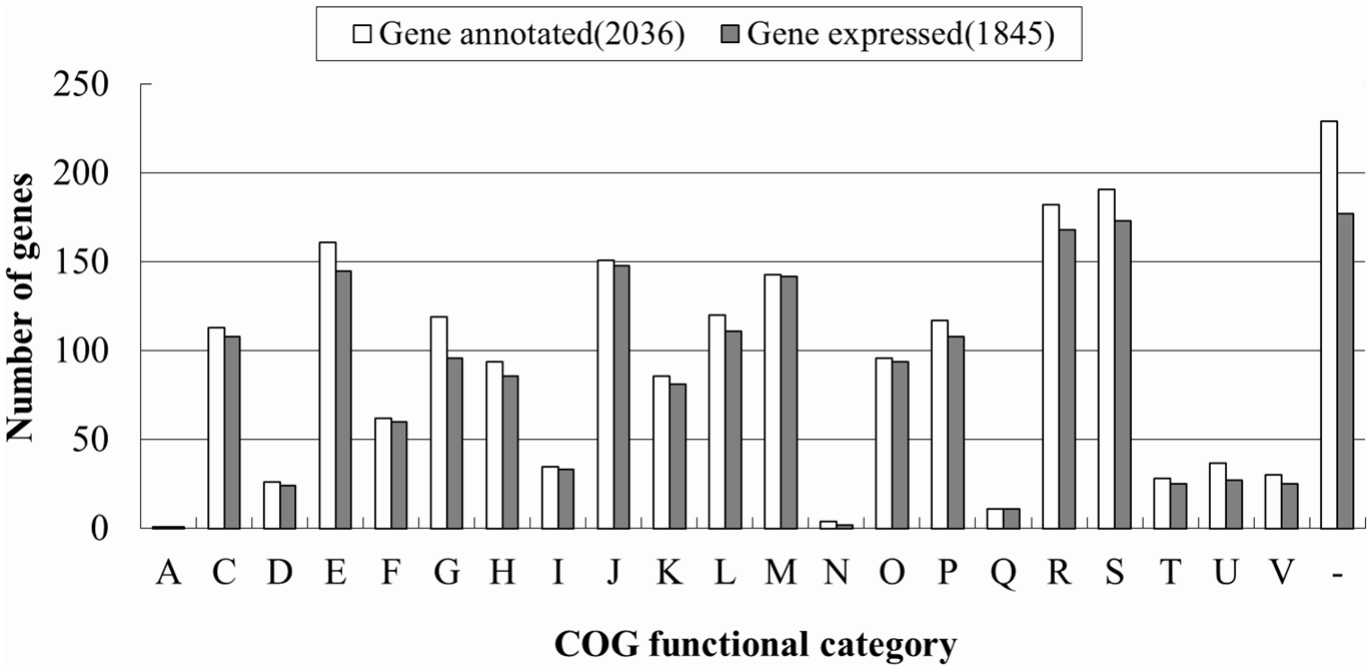
Clusters of orthologous groups (COG) classification of the expressed *A*. *pleuropneumoniae* genes. Bars in dark gray indicate numbers of expressed genes (n = 1845); while bars in white indicate numbers of all protein-coding genes (n = 2,036) in *A*. *pleuropneumoniae* JL03. COG categories: J, translation; A, RNA processing and modification; K, transcription; L, DNA replication, recombination and repair; D, cell division and chromosome partitioning; V, defense mechanisms; T, signal transduction; M, cell wall/membrane biogenesis; U, intracellular trafficking, secretion and vesicular transport; O, posttranslational modification, protein turnover and chaperones; C, energy production and conversion; G, carbohydrate transport and metabolism; E, amino acid transport and metabolism; F, nucleotide transport and metabolism; H, coenzyme metabolism; I, lipid metabolism; P, inorganic ion transport and metabolism; Q, secondary metabolites biosynthesis, transport and catabolism; R, general functional prediction only; S, function-unassigned; -, unknown proteins not in the COG categories.

**Fig 2:**
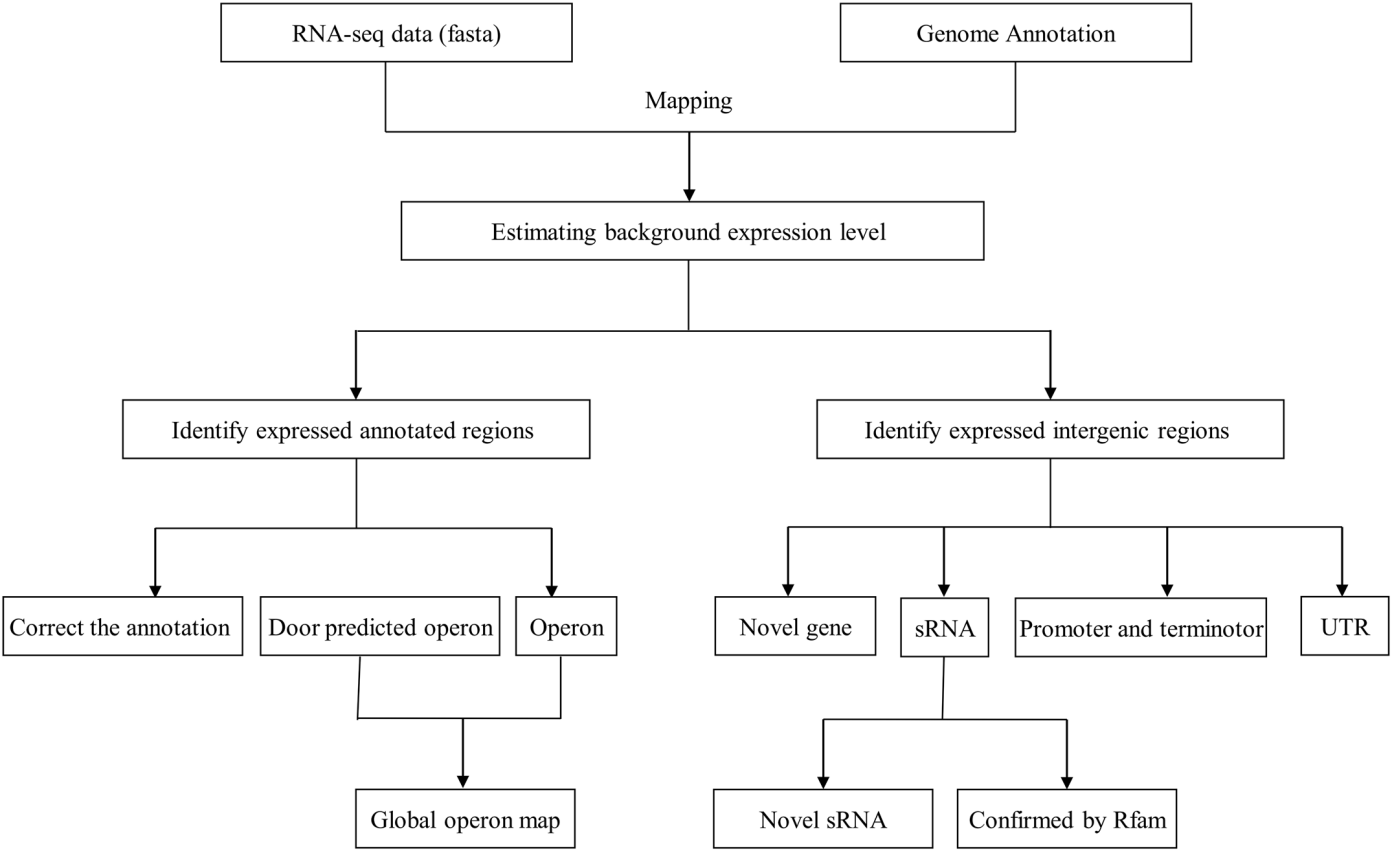
Worlflow of RNA-Seq data analysis. RNA-seq reads were mapped to the genome using the alignment program BWA. Combining with the existing annotation, expressed annotated regions and expressed intergenic regions are generated by using SAMTools and Perl scripts. Novel protein coding genes, UTRs and sRNAs are identified.

The complete genome sequence of the *A*. *pleuropneumoniae* JL03 was published in 2008 [GenBank: CP000687] [12]. The 2.24 megabase circular genome carries 2,147 computationally predicted genes of which 2,036 are protein coding with a 41.23% G+C content. To obtain a global view of the *A*. *pleuropneumoniae* transcriptome at single-nucleotide resolution, we sequenced the transcriptome of the wild type *A*. *pleuropneumoniae* JL03 strain using RNA-Seq. RNA was isolated from bacteria harvested at the mid-exponential growth phase (S2 Fig). After removing the low-quality reads, a total of 38,568,297 reads with an average length of 90 bp were obtained. The total length of the reads was about 3.5 Gb, representing about a more than 1700-fold coverage of the *A*. *pleuropneumoniae* genome. Totally, 93.22% reads were mapped onto the reference DNA sequence. After estimation of the background expression level using the criteria described in methods, the cutoff value was calculated as 71 reads/bp. Based on this cutoff, we identified 1933 (90.03%) out of the 2147 predicted genes and 1845 out of the 2036 protein-coding genes which were expressed. The functions of the expressed genes were distributed across all categories of COG database [28] (Fig 1). We also identified 1221 previously un-annotated expressed intergenic regions (EIRs) with a minimum length of 30 bp. The transcriptome analysis workflow is represented in Fig 2.

### Identification and characterization of novel genes

**Fig 3:**
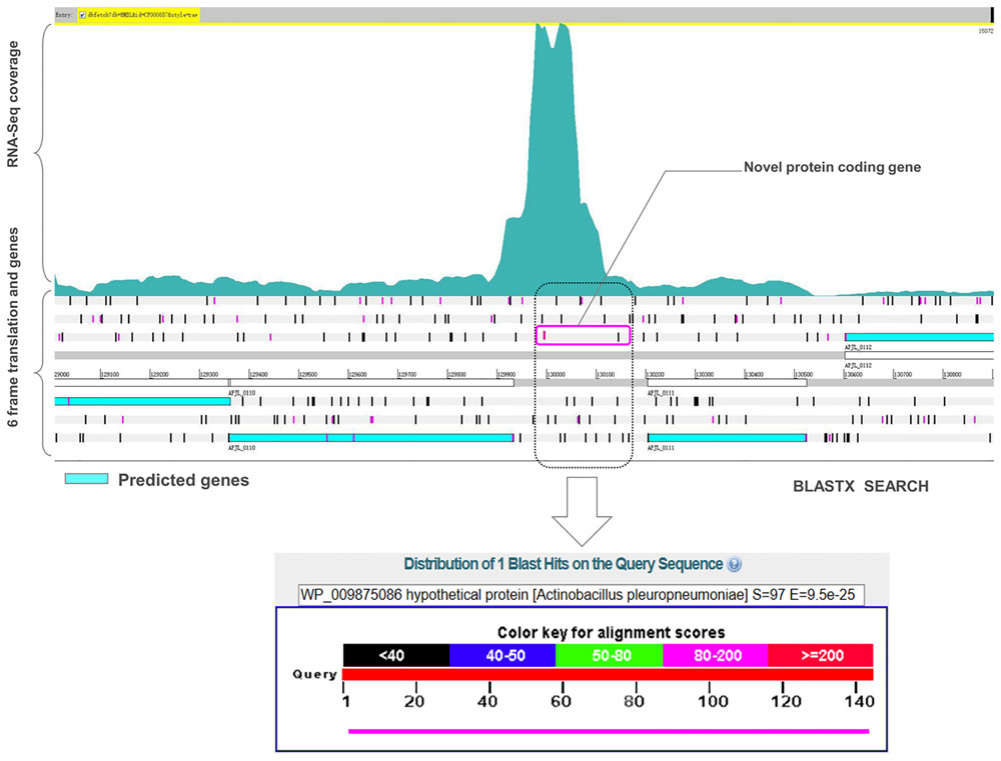
Identification of a novel protein coding gene within the single-nucleotide resolution transcriptome map of *A*. *pleuropneumoniae*. Genomic loci and transcriptional profiles were visualized in the Artemis genome browser. The figure shows identification of a novel protein coding gene ‘‘APP-P04” using RNA-seq data. A BLASTX search of the ORF shows the homology (similarity 100%, sequence coverage 100%) to a hypothetical protein in *A*. *pleuropneumoniae*.

Overall, 32 novel protein coding regions were identified according to the transcriptome map (Table 1). The average length of proteins encoded by these regions was around 47 amino acids (ranged from 26 to 90 amino acids). 81.3% of the proteins encoded by the novel genes were annotated as hypothetical proteins, with only six proteins having predicted functions, a DNA methylase, a Leucyl-tRNA synthetase, a phosphoribosylformylglycinamidine cyclo-ligase, two transposases and a naphthoate synthase (Table 1). Fig 3 shows an example of a novel gene named ‘‘APP-P04” encoding a protein that is identical to (100% identities and 100% coverage) a hypothetical protein already annotated in other *A*. *pleuropneumoniae* genomes.

**Table 1:** Novel protein coding genes identified in the *A*. *pleuropneumoniae* JL03 genome.

ID	Strand	Length (bp)	Start	End	Top BLASTX Hit (Protein Accession No.)	Identity (%)	Query coverage (%)	Annotation
APP-P01	-	159	14888	15046	EFX91114.1	79	81	hypothetical protein HMPREF0027_1817 [*Actinobacillus ureae* ATCC 25976]
APP-P02	+	141	748863	749003	EFM92177.1	98	97	hypothetical protein appser6_7570 [*Actinobacillus pleuropneumoniae* serovar 6 str. Femo]
APP-P03	+	123	126371	126493	EFM86287.1	100	97	hypothetical protein appser1_1260 [*Actinobacillus pleuropneumoniae* serovar 1 str. 4074]
APP-P04	+	144	130000	130143	WP_041603459.1	100	100	hypothetical protein [*Haemophilus ducreyi* strain ATCC 33921]
APP-P05	-	96	165125	165220	EFM86331.1	100	100	hypothetical protein appser1_1700 [*Actinobacillus pleuropneumoniae* serovar 1 str. 4074]
APP-P06	-	138	215622	215759	ACE60849.1	100	97	hypothetical protein APP7_0197 [*Actinobacillus pleuropneumoniae* serovar 7 str. AP76]
APP-P07	+	123	348715	348837	EFM86224.1	100	97	hypothetical protein appser1_3330 [*Actinobacillus pleuropneumoniae* serovar 1 str. 4074]
APP-P08	-	114	564175	564288	AFU19023.1	100	94	hypothetical protein ASU2_04425 [*Actinobacillus suis* H91-0380]
APP-P09	-	120	753874	753993	EFM85822.1	97	95	hypothetical protein appser1_7490 [Actinobacillus pleuropneumoniae serovar 1 str. 4074]
APP-P10	+	138	780517	780654	AGQ24929.1	100	91	DNA methylase [*Mannheimia haemolytica* D153]
APP-P11	-	78	965335	965412	EFL78484.1	100	100	hypothetical protein APP2_1809 [*Actinobacillus pleuropneumoniae* serovar 2 str. 4226]
APP-P12	-	150	977266	977415	EFM96564.1	81	64	Leucyl-tRNA synthetase [*Actinobacillus pleuropneumoniae* serovar 10 str. D13039]
APP-P13	-	120	1005003	1005122	EFM94226.1	100	97	hypothetical protein appser9_9720 [*Actinobacillus pleuropneumoniae* serovar 9 str. CVJ13261]
APP-P14	+	114	1035429	1035542	EFM85421.1	100	97	hypothetical protein appser1_10430 [*Actinobacillus pleuropneumoniae* serovar 1 str. 4074]
APP-P15	+	162	1051786	1051946	EFM91996.1	96	98	hypothetical protein appser6_10890 [*Actinobacillus pleuropneumoniae* serovar 6 str. Femo]
APP-P16	-	123	1113719	1113841	EFM85479.1	100	97	hypothetical protein appser1_11010 [*Actinobacillus pleuropneumoniae* serovar 1 str. 4074]
APP-P17	-	108	1229502	1229609	ACE61803.1	42	91	phosphoribosylformylglycinamidine cyclo-ligase [*Actinobacillus pleuropneumoniae* serovar 7 str. AP76]
APP-P18	-	219	1457550	1457768	WP_039709255.1	100	98	hypothetical protein [*Actinobacillus pleuropneumoniae*]
APP-P19	+	270	1457617	1457886	AFU19571.1	72	98	hypothetical protein ASU2_07165 [*Actinobacillus suis* H91-0380]
APP-P20	-	84	1459981	1460064	EFM85119.1	100	73	hypothetical protein appser2_13220 [*Actinobacillus pleuropneumoniae* serovar 2 str. S1536]
APP-P21	+	201	1492300	1492500	EFX92265.1	78	47	hypothetical protein HMPREF0027_0694 [*Actinobacillus ureae* ATCC 25976]
APP-P22	-	138	1519116	1519253	EFM85119.1	100	73	hypothetical protein appser1_14900 [*Actinobacillus pleuropneumoniae* serovar 1 str. 4074]
APP-P23	-	144	1627977	1628120	EFM91430.1	100	97	hypothetical protein appser6_16080 [*Actinobacillus pleuropneumoniae* serovar 6 str. Femo]
APP-P24	-	111	1660694	1660804	AEC16624.1	47	91	putative transposase [*Gallibacterium anatis* UMN179]
APP-P25	-	123	1706458	1706580	EFM84885.1	100	97	hypothetical protein appser1_16470 [*Actinobacillus pleuropneumoniae* serovar 1 str. 4074]
APP-P26	-	128	1753350	1753477	EQA09464.1	96	63	naphthoate synthase [*Haemophilus parasuis* D74]
APP-P27	-	138	1928943	1929080	EFM86903.1	100	100	hypothetical protein appser2_17520 [Actinobacillus pleuropneumoniae serovar 2 str. S1536]
APP-P28	+	162	1933706	1933867	EDN73192.1	64	70	hypothetical protein MHA_0204 [*Mannheimia haemolytica* PHL213]
APP-P29	+	214	1995103	1995316	AEC16624.1	99	47	putative transposase [*Gallibacterium anatis* UMN179]
APP-P30	+	207	2026720	2026926	KDD80553.1	68	49	hypothetical protein HPS41_03095, partial [*Haemophilus parasuis* ST4-1]
APP-P31	+	126	2062043	2062168	EFM84509.1	98	97	hypothetical protein appser1_19900 [*Actinobacillus pleuropneumoniae* serovar 1 str. 4074]
APP-P32	+	111	2164127	2164237	EFM84431.1	100	97	hypothetical protein appser1_20960 [*Actinobacillus pleuropneumoniae* serovar 1 str. 4074]

### Refinement of the existing genome annotation

**Fig 4:**
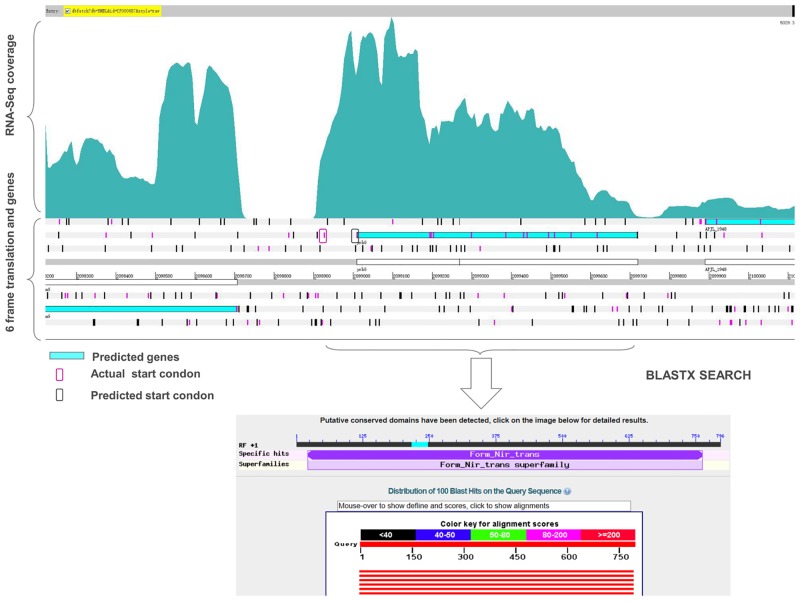
Correction made to the start site of a gene. Visualization of the single-nucleotide resolution transcriptome map shows the transcription of upstream of the gene ‘‘APJL_1947”. The predicted and actual start codons within the ORF are marked.

Based on the RNA-seq data, we obtained the transcriptional evidence of the existing genome annotation. When visualized in Artemis, the transcriptome map helped to identify incorrect annotation of start codons and real length of the annotated proteins. Some annotated proteins had the same termination codons, but the transcriptional start sites identified in this study are different from the annotated start codons, which suggest that the previously annotated start codons are incorrect. This enabled us to correct the start site for 35 genes based on the current genome annotation (Table 2). Then, a comparison with the similar proteins in related species and ORF search were performed to finally confirm the gene boundaries (Table 2). An example is presented for the gene ‘‘APJL_1947”, annotated as ‘‘putative formate-nitrite transporter, *YrhG*” (Fig 4). The actual start codon site was found to be 84 bp upstream of the previous annotated site.

**Table 2:** Corrections made to the existing genome annotation.

Gene id	Previous location (Start-End)	Corrected location (Start-End)	Strand
APJL_0019	20445–21500	20262–21500	+
APJL_0022	24103–24792	23998–24792	+
APJL_0040	42108–43754	42069–43754	+
APJL_0076	85484–86218	85484–86341	-
APJL_0181	200430–200951	200430–201254	-
APJL_0188	207972–209312	207972–209409	-
APJL_0194	213857–214846	213857–214957	-
APJL_0295	316686–317570	316566–317570	-
APJL_0486	537367–538905	537148–538905	+
APJL_0500	550347–553106	550233–553106	+
APJL_0519	565329–566192	565197–566192	+
APJL_0617	670415–670906	670415–670996	-
APJL_0669	731972–733252	731972–733378	-
APJL_0734	804664–805113	804541–805113	+
APJL_0743	811177–811851	811177–811882	-
APJL_0838	926340–927020	926340–927185	-
APJL_1170	1291694–1292098	1291628–1292098	+
APJL_1179	1302358–1303407	1302358–1303479	-
APJL_1230	1351089–1353617	1351089–1353755	-
APJL_1291	1419511–1420647	1419511–1420770	-
APJL_1320	1447455–1447955	1447455–1448069	-
APJL_1343	1473151–1474092	1473079–1474092	+
APJL_1347	1481231–1481647	1481231–1481752	-
APJL_1363	1501745–1502311	1501745–1502398	-
APJL_1384	1519258–1520565	1519258–1520709	-
APJL_1510	1660978–1661679	1660978–1661838	-
APJL_1590	1748017–1749288	1748017–1749402	-
APJL_1598	1756635–1758095	1756635–1758278	-
APJL_1617	1774727–1775452	1774655–1775452	+
APJL_1658	1811042–1811797	1810898–1811797	+
APJL_1761	1916914–1917699	1774655–1775452	+
APJL_1947	2099009–2099719	2098925–2099719	+
APJL_2009	2160426–2161499	2160132–2161499	+
APJL_2035	2180950–2181522	2180776–2181522	+
APJL_2074	2218634–2219587	2218511–2219587	+

### Identification of small non-coding RNAs and UTRs

**Fig 5:**
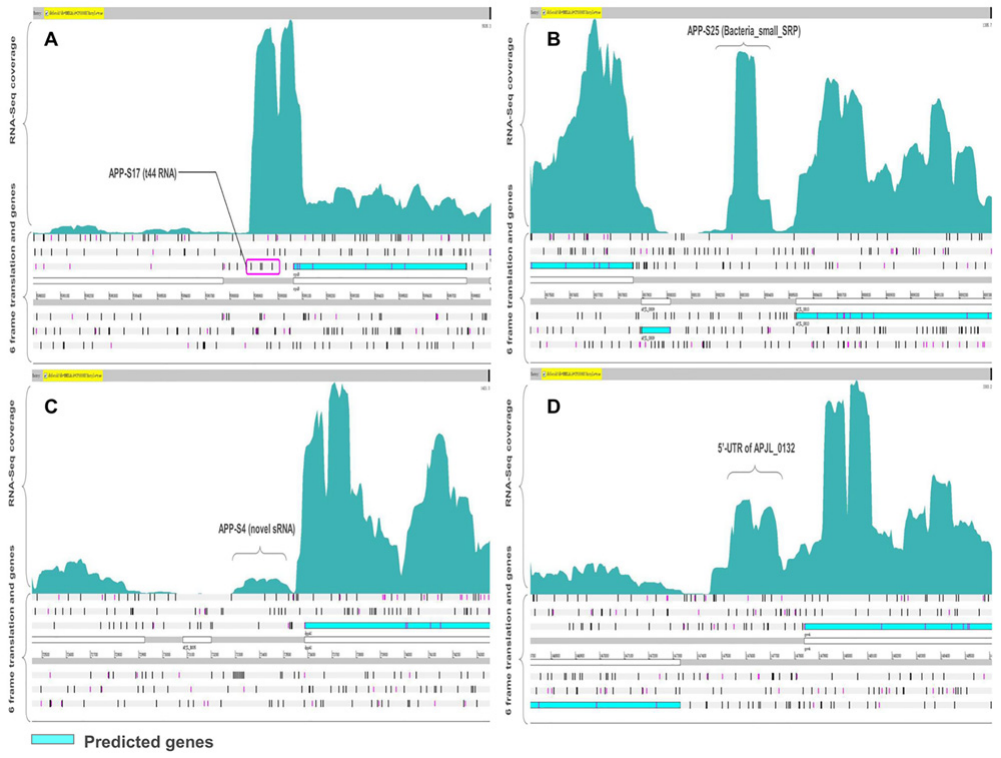
Identification of potential sRNA and UTR. (A) Identification of a well conserved sRNA ‘‘APP-S17” in the 5’ UTR region of the gene ‘‘APJL_0559”, which was classified as t44 RNA by Rfam. (B) Identification of a highly expressed sRNA ‘‘APP-S25” annotated by Rfam as bacterial signal recognition particle RNA. (C) Identification of a novel sRNA ‘‘APP-S4” in the intergenic region of *A*. *pleuropneumoniae* JL03 genome. (D) Identification of the 5’UTR region of the gene ‘‘APJL_0132”.

Bacterial small RNAs (sRNAs) are short (30–500 bp) untranslated transcripts and often act as regulators of gene expression through a variety of mechanisms [31–33]. The identification of sRNA in the genome is the foundation to understand their role in modulating bacterial physiology and virulence [34]. In this study, we identified 51 putative sRNAs in the *A*. *pleuropneumoniae* JL03 genome (Table 3). The average length of the identified novel sRNA was approximate 100 bp and ranged from 30 to 303 bp. All identified sRNA had a promoter or terminator adjacent to their locus which can increase the confidence of the sRNA [35, 36]. By comparing with the sRNA sequences in the Rfam database, 11 sRNAs of *A*. *pleuropneumoniae* were homologous to well characterized sRNAs in other species, such as t44 RNA and Bacteria_small_SRP (Bacterial small signal recognition particle RNA) (Fig 5A and 5B). The other identified functional sRNAs categories included 6S, *cspA* mRNA 5' UTR, GcvB, MOCORNA, Glycine riboswitch, Alpha_RBS and His_leader. On the other hand, 40 sRNAs with no matches in the Rfam database could be predicted as novel sRNAs. Fig 5C shows the depth of coverage for one of the identified novel sRNA ‘‘APP-S4”.

**Table 3:** sRNAs identified in *A*. *pleuropneumoniae* JL03.

ID	Start^#^	End^#^	Length (nt)	Promoter	Terminator	Flanking gene (left)	Flanking gene (right)	Rfam annotation	Conservation across other genome*
APP-S1	43810	43854	45	Y	-	APJL_0040(+)	APJL_0041(+)	-	A
APP-S2	69850	69943	94	Y	-	APJL_0062(-)	APJL_0062(+)	-	A
APP-S3	69955	70012	58	Y	-	APJL_0062(-)	APJL_0062(+)	-	B
APP-S4	73341	73502	162	Y	-	APJL_0062(-)	APJL_0062(+)	-	A
APP-S5	128399	128496	98	Y	Y	APJL_0108(-)	APJL_0109(-)	-	A
APP-S6	129934	130203	270	Y	-	APJL_0110(-)	APJL_0111(-)	6S	C
APP-S7	136144	136180	36	-	Y	APJL_0118(+)	APJL_0119(-)	-	A
APP-S8	136390	136532	132	Y	-	APJL_0119(-)	APJL_0120(+)	cspA	C
APP-S9	147458	147839	381	-	Y	APJL_0131(-)	APJL_0132(+)	GcvB	B
APP-S10	184303	184395	93	Y	-	APJL_0165(-)	APJL_0166(+)	-	A
APP-S11	184404	184496	93	Y	-	APJL_0165(-)	APJL_0166(+)	-	A
APP-S12	193258	193351	94	Y	Y	APJL_0174(-)	APJL_0175(+)	-	A
APP-S13	283501	283565	65	Y	-	APJL_0261(+)	APJL_0262(-)	-	A
APP-S14	383608	383698	91	Y	Y	APJL_0358(-)	APJL_0359(-)	-	A
APP-S15	466389	466478	90	-	Y	APJL_0425(-)	APJL_0426(+)	-	A
APP-S16	517137	517215	79	-	Y	APJL_0468(+)	APJL_0471(-)	-	A
APP-S17	598881	599063	182	-	Y	APJL_0553(+)	APJL_0559(+)	t44	B
APP-S18	703124	703153	30	Y	Y	APJL_0644(-)	APJL_0645(-)	-	C
APP-S19	753851	754045	195	-	Y	APJL_0686(-)	APJL_0687(-)	MOCO_RNA_motif	B
APP-S20	755351	755515	164	-	Y	APJL_0687(-)	APJL_0688(+)	MOCO_RNA_motif	B
APP-S21	765539	765588	50	Y	Y	APJL_0696(-)	APJL_0697(-)	-	B
APP-S22	773794	773833	40	-	Y	APJL_0700(-)	APJL_0703(-)	-	A
APP-S23	783905	783980	76	Y	-	APJL_0707(-)	APJL_0708(+)	-	A
APP-S24	801903	801952	50	Y	Y	APJL_0730(+)	APJL_0731(-)	-	B
APP-S25	888250	888433	184	Y	-	APJL_0809(-)	APJL_0810(-)	Bacteria_small_SRP	C
APP-S26	908467	908556	90	Y	-	APJL_0825(-)	APJL_0826(+)	-	A
APP-S27	1004946	1005007	62	Y	-	APJL_0908(+)	APJL_0909(+)	-	A
APP-S28	1144702	1144793	92	Y	Y	APJL_1031(-)	APJL_1032(-)	-	A
APP-S29	1211704	1211736	33	Y	Y	APJL_1093(-)	APJL_1094(-)	-	D
APP-S30	1227906	1227961	56	Y	Y	APJL_1107(-)	APJL_1108(-)	-	A
APP-S31	1236854	1236954	101	Y	Y	APJL_1117(+)	APJL_1118(+)	-	A
APP-S32	1239160	1239199	40	Y	Y	APJL_1119(+)	APJL_1120(+)	-	C
APP-S33	1253148	1253181	34	Y	Y	APJL_1129(+)	APJL_1130(+)	-	B
APP-S34	1269545	1269848	303	Y	-	APJL_1146(-)	APJL_1147(-)	Glycine	C
APP-S35	1304170	1304219	50	-	Y	APJL_1180(-)	APJL_1181(-)	-	A
APP-S36	1327989	1328023	35	-	Y	APJL_1205(+)	APJL_1206(-)	-	B
APP-S37	1375766	1375806	41	-	Y	APJL_1251(+)	APJL_1252(+)	-	B
APP-S38	1431012	1431043	32	Y	-	APJL_1305(-)	APJL_1306(+)	-	B
APP-S39	1471055	1471094	40	Y	-	APJL_1340(+)	APJL_1341(+)	-	A
APP-S40	1575463	1575524	62	Y	Y	APJL_1433(+)	APJL_1434(+)	-	A
APP-S41	1591719	1591803	85	Y	-	APJL_1447(-)	APJL_1448(+)	-	A
APP-S42	1610164	1610233	70	Y	-	APJL_1463(-)	APJL_1464(+)	-	A
APP-S43	1742806	1742919	114	Y	-	APJL_1585(-)	APJL_1586(+)	-	A
APP-S44	1810752	1810833	82	Y	-	APJL_1657(-)	APJL_1658(+)	-	A
APP-S45	1861523	1861613	91	-	Y	APJL_1704(+)	APJL_1705(+)	-	A
APP-S46	1893513	1893575	63	Y	-	APJL_1732(-)	APJL_1733(+)	-	A
APP-S47	1968939	1969074	136	Y	Y	APJL_1816(+)	APJL_1817(+)	Alpha_RBS	C
APP-S48	1994835	1994869	35	Y	-	APJL_1847(-)	APJL_1848(+)	-	B
APP-S49	2032687	2032872	186	Y	-	APJL_1880(+)	APJL_1881(-)	-	A
APP-S50	2054874	2054924	51	Y	Y	APJL_1898(+)	APJL_1899(+)	-	A
APP-S51	2212991	2213203	212	-	Y	APJL_2068(-)	APJL_2069(+)	His_leader	B

^#^The start and end represents the boundaries of identified TAR(transcriptionally active region)which is a potential sRNA region.

*sRNA sequences conserved in; A- *Actinobacillus pleuropneumoniae*. B- *Actinobacillus*. C- *Pasteurellales*. D-across distant bacterial species.

To study the conservation of identified sRNAs of *A*. *pleuropneumoniae*, the sequences of all predicted sRNA were searched against the non-redundant, nucleotide database at NCBI. Thirty of the sRNAs were highly conserved (>95% identity with 100% coverage) only in *A*. *pleuropneumoniae*. A set of 20 sRNAs were conserved in the related *Pasteurellaceae* family including *M*. *varigena*, *M*. *haemolytica*, *A*. *suis*, *B*. *trehalosi and A*. *lignieresii*. Only 1 sRNA was also present in the species far from *A*. *pleuropneumoniae*, such as in *Mycoplasma pulmonis* and *Vibrio*.

The bacterial untranslated regions (UTRs) contain regulatory elements to control gene expression [37, 38]. The 3’-UTRs can be a source of regulatory RNAs in bacteria [39]. RNA-seq allows the analysis of 5’and 3’-UTR regions. We identified 5’UTR for 715 annotated genes and 3’UTR for 384 annotated genes in *A*. *pleuropneumoniae* JL03 (S1 Table). One 5’UTR in the transcriptome map is shown in Fig 5D. With further analysis of the identified sRNAs, 11 predicted sRNA were found to be located in the immediate vicinity of a protein-coding region which might be processed from the 5’- or 3’-UTR of the corresponding mRNA.

### Characterization of Operon

**Fig 6:**
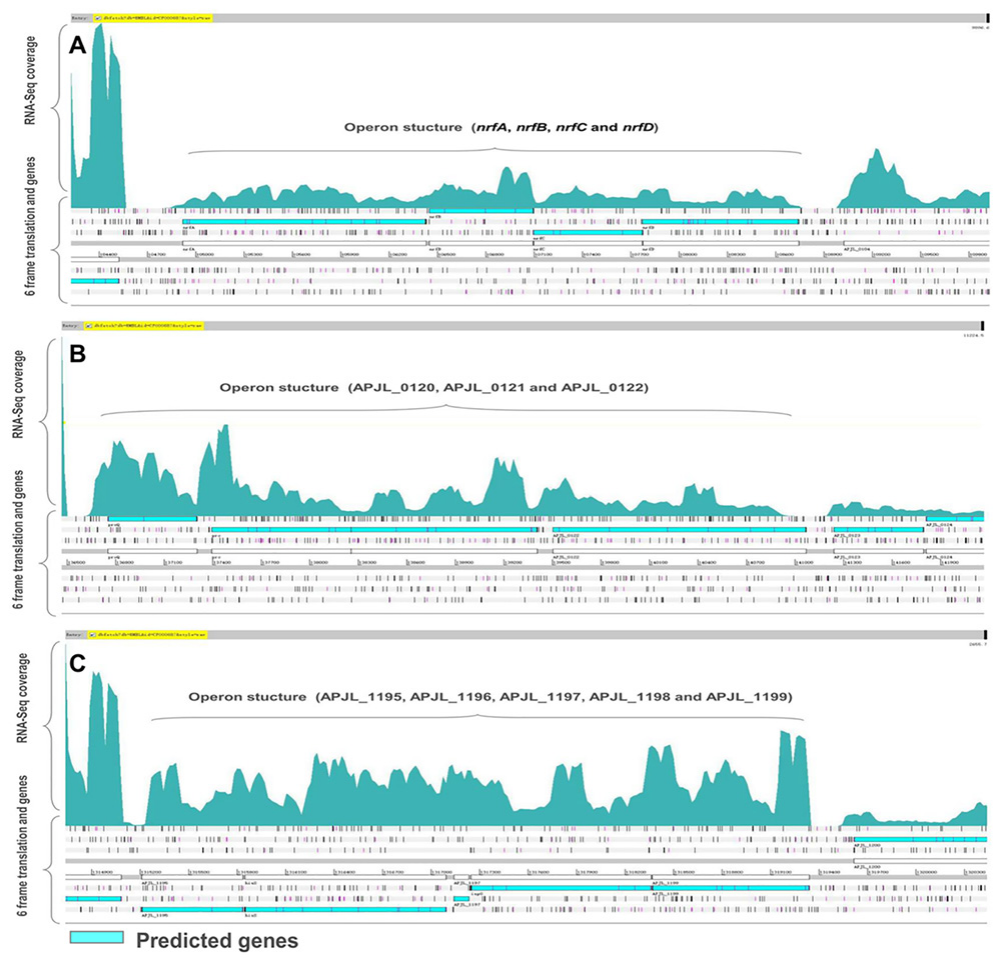
Single-nucleotide resolution transcriptome map revealing the operon structures of *A*. *pleuropneumoniae* JL03. The operons predicted by RNA-seq and the operons predicted by DOOR were compared. (A) An operon common to both DOOR and RNA-Seq. (B) An operon identified based on RNA-seq data but not previously predicted by DOOR. (C) An operon identified based on RNA-seq data having different sizes with the one predicted by DOOR.

By using the transcriptome map, the operon structures can be determined at the genome scale [20, 40]. To evaluate the accuracy of the operon based on RNA-seq data, we also compared our identified co-expressed genes and operon structures with computationally predicted operons in DOOR. Based on RNA-seq data, 840 co-expressed pairs of genes that could be organized into 351 operons were identified. DOOR has predicted 860 co-expressed genes forming 460 multi-gene operons in *A*. *pleuropneumoniae* JL03 (S2 and S3 Tables). Through the comparative analysis, 213 operons found in this study are identical to the predicted operons in DOOR and 104 operons are different. Thirty-four operons based on RNA-seq data are not previously predicted by DOOR (S3 Table). Three typical operon structures visualized on the transcriptome map are displayed in Fig 6. Operon “APP-O24” consisting of four genes, the *nrfA*, *nrfB*, *nrfC* and *nrfD* encoding nitrite reductase subunits, was predicted by DOOR and also confirmed by RNA-seq (Fig 6A). A new operon “APP-O28” comprised of three genes (APJL_0120, APJL_0121 and APJL_0122) was identified based on RNA-seq data but not predicted in DOOR (Fig 6B). Fig 6C shows the operon “APP-O206” which contains five genes (APJL_1195, APJL_1196, APJL_1197, APJL_1198 and APJL_1199) and is different with the structure in the DOOR database. The overlap number of co-expressed gene pairs between RNA-Seq based and DOOR-based was 658 which validated the computational gene-pair predictions (S2 Table). The 182 new gene pairs that are co-expressed but not predicted by DOOR well complemented the operon structures of *A*. *pleuropneumoniae* (S2 Table).

## Discussion

Genome annotations can bridge the gap between sequence and biology, providing a framework to understand the biological process of various bacteria, archaea and eukaryotic species. Comprehensive and accurate annotation of the genomic sequence is crucial for analysis and investigations of functional genes and regulatory elements at systems level. Previous genome sequencing has the limitations that it can not display real transcription regions and non-coding transcripts, which leads to the mis-annotations of genes and neglected numerous functional elements in the intergenic regions, especially small non-coding RNAs (sRNAs). With the accumulation of sequence information and the improvements in bioinformatic techniques, original genome annotations need to be revised to make them more accurate and useful to explore gene functions and regulations. To acquire complete and well described genome annotation, plenty of studies have worked on genome re-annotation with various methods in eukaryotes and prokaryotes [41–43]. Both computational and experimental approaches (such as computational methods using various algorithms [44, 45], comparative genomics [46, 47] and whole genome microarrays [48, 49]) have corrected many predicted genes and revealed numerous novel genes. However, identification and demarcation of the boundaries of all the functional elements such as untranslated regions (UTRs), regulatory regions, and operon structures are still big challenges. To overcome these difficulties, whole transcriptome RNA sequencing (RNA-seq) has been used to improve the genome annotations [50, 51]. In comparison to microarray, RNA-Seq has the advantages on increased dynamic range and low background noise, which has been demonstrated to be effective in the correction of gene annotation [10, 36], discovery of novel transcripts and non-coding RNAs [52], lowly expressed transcripts [53] and accurate operon structures [54]. For example, RNA-Seq based transcriptome analysis identified 14 novel protein coding regions, 44 sRNAs and 518 expressed operons in *M*. *haemolytica* PHL213[36].

The *A*. *pleuropneumoniae* JL03 genome was sequenced and published in 2008 which provided the foundation for all subsequent analysis of the *A*. *pleuropneumoniae* genome and gene functions [55–58]. But the functional and structural annotations of genomes are inaccurate based on the DNA level. The regulatory elements in the genome have not yet been fully elucidated. In this study, we combined RNA-seq with bioinformatical methods to re-annotate and analyze the *A*. *pleuropneumoniae* JL03 genome at the transcriptome level. A single resolution transcription map of the *A*. *pleuropneumoniae* JL03 genome was generated which enabled us to re-annotate the existing genome. Ninety-four percent of the RNA-seq reads mapped to the *A*. *pleuropneumoniae* JL03 genome, which is comparable with the other RNA-seq studies. Ninety-four percent cDNA reads were assignable to the genome of *Bacillus anthracis* [9], 95% in *Burkholderia cenocepacia* [59] and 94% in *Histophilus somni* [10], which illuminates the appropriateness of using RNA-seq for bacterial transcriptomic studies. The data analysis revealed 32 novel protein coding genes, with the average length around 47 amino acids. These genes were missed in previous annotations possibly due to their relatively short size. Most of the proteins have not been reported or predicted functions. During the genomic sequence-based gene annotation, it is difficult to predict the precise start codon of gene. Through the transcriptome map, we identified 35 genes with incorrect annotation of start codons in the genome. Based on BLAST searching against homologous genes in other bacterial species, we confirmed the actual start codon and defined the actual boundaries. Consequently, the proteins encoded by these genes are longer than previously predicted. Identification of the actual start codon is crucial for understanding and characterizing the proteome. Future work can be done on the protein level to further validate the new annotations and investigate the function of the newly defined proteins.

In bacteria, small regulatory RNAs (sRNAs) play a pivotal role in regulation of the gene expression, controlling a variety of adaptation processes in response to changes in the environment. Although the genome has been sequenced several years ago, no information about sRNAs is available in *A*. *pleuropneumoniae*. Because of the small size and low expression level, sRNAs are hard to detect using traditional screening strategy. On account of lack of defined sequence features, computational approach for sRNA prediction is not powerful enough. Previous studies have shown the power of RNA-seq in bacterial sRNA identification [9, 60]. In this work, we identified 51 putative sRNAs that were transcribed in mid-log phase of *A*. *pleuropneumoniae*. We found that 10 of the 51 sRNAs were homologous to characterized sRNAs. For instance, APP-S17 was classified as T44 RNA, which has been identified in several species of enteric bacteria [48]. The function of this RNA is still unknown. APP-S25 was classified as bacterial signal recognition particle RNA, which is the RNA component of the signal recognition particle (SRP) ribonucleoprotein complex. These results validate our approach. Interestingly, the 6S RNA, a global regulator of transcription in bacteria, was found in our study with high transcription levels (APP-S6). In *E*. *coli*, 6S RNA alters the transcription of most genes in stationary phase. During this phase, 6S RNA accumulates in cells; loss of 6S RNA leads to decreased survival compared to wild-type cells [61]. However, 6S RNA accumulation profiles also vary, suggesting diversity in the time and the location at which it exert effects [62]. Recent studies in *E*. *coli* suggest 6S RNA may contribute significantly to transcriptional regulation even when levels are not maximal, such as in exponential phase when 6S RNA is only one-tenth as abundant as in stationary phase [62]. In addition to down-regulating some housekeeping genes, 6S RNA has been shown to up-regulate expression of some σ^S^-dependent genes in Gram-negative bacteria [62]. Taken together, APP-S6 might play roles in regulating the expression of an abundance of genes in exponential phase in *A*. *pleuropneumoniae*. We also identified APP-S9 which was classified as GcvB RNA. The GcvB RNA has been found in a range of bacteria such as *E*. *coli* [63], *Y*. *pestis* [64], *H*. *somni* [10] and *Salmonella* [65]. In *E*. *coli* and *Salmonella* species, the GcvB RNA has been shown to regulate a large number of genes which are involved in the amino acid transport systems and amino acid biosynthesis [63, 65, 66]. In *E*. *coli*, transcription of the GcvB RNA is activated by the adjacent *gcvA* gene and regulates genes by acting as an antisense binding partner of the mRNAs for each regulated gene [63]. APP-S9 was located in the upstream of *gcvA* (APJL_0132) which was annotated as DNA-binding transcriptional activator. Taken together, we hypothesize that APP-S9 may be activated by *gcvA* (APJL_0132) and have regulatory activity in the amino acid transport systems and amino acid biosynthetic genes in *A*. *pleuropneumoniae*. The other 41 novel identified sRNAs may play regulatory roles in basic life and/or virulence as described in other bacteria [67, 68]. Among the identified sRNAs, 30 of which were highly conserved (>95% identity with 100% coverage) only in *A*. *pleuropneumoniae* implying these sRNA are *A*. *pleuropneumoniae*-specific. Only 1 sRNA is present in the species far from *Pasteurellceae* family. This sRNA may be responsible for the basic survival of many species. The sRNAs present in different species may be functional in adaptation to different environmental conditions.

RNA-Seq data provides information to identify the UTRs of annotated genes. Until now, the mapping of UTRs has not been performed on *A*. *pleuropneumoniae* which can discover regulatory elements in these regions such as riboswitches, RNA thermometers or small leader peptides. We identified 5’-UTR of 715 annotated genes and 3’-UTR of 384 annotated genes in *A*. *pleuropneumoniae* JL03. Most of the identified 5’-UTRs (72%) were very short with the size less than 100 bp, which is similar to other bacterial, such as 75% of the 5’-UTRs for annotated genes were shorter than 100 bp in *C*. *glutamicum* ATCC 13032 [54, 69]. With further analysis, we found that 9 sRNAs were located in the UTR regions. APP-S20 has a MOCO RNA motif which is presumed to be a riboswitch that binds to molybdenum cofactor or related tungsten cofactor [70]. In *E*. *coli*, the riboswitches upstream of *moaA* gene regulate translation of the *moaABCDE* operon by blocking ribosome-binding sites [70]. APP-S20 was found 155 bp upstream of *moaA* (APJL_0688) which was annotated as molybdenum cofactor biosynthesis protein A, implying its regulatory role in molybdenum cofactor biosynthesis. Additionally, one RNA thermometer (APP-S8) was observed within the 5’-UTR of *cspC* (APJL_0119). The gene *cspC* encodes a cold shock-like protein. Cold shock proteins are stress induced regulators which is functional in response to low temperature and also in normal conditions [71, 72]. The *cspC* was repressed after acute infection of *A*. *pleuropneumoniae* [73] and after exposure to host hormones [74], suggesting that they are involved in infection of *A*. *pleuropneumoniae*. In *E*. *coli*, the *cspA* thermosensor acts as an RNA thermometer, regulating the expression of *cspA* in response to temperature [75]. Hence, APP-S8 is possible a regulator of *cspC*. A recent study has documented that Gram-negative bacteria contain a conserved motif in their 5’-leader sequences [76]. In *A*. *pleuropneumoniae*, we found a small leader peptide (APP-S51) within 5’-UTRs of the operon involved in histidine synthesis (APJL_2069/*hisG*, APJL_2070/*hisD*, APJL_2071/*hisC2*, APJL_2072/- and APJL_2073/*hisB*). These leader peptides are possibly involved in transcription attenuation of the target genes dependent on the amino acid level [77]. In conclusion, these results imply that UTRs may play regulatory roles in the complicated process of gene expression of *A*. *pleuropneumoniae*.

For prokaryotic organisms, identification of operon structures at the genome scale is significant in studying the gene function and the regulatory networks. Based on the RNA-seq data, we identified 351 expressed operons covering 1,230 genes of *A*. *pleuropneumoniae*. The majority of the identified operons contain 2–9 genes, which is a general trend in other previously reported bacteria [6, 54]. Furthermore, we compared our prediction results with 460 predicted operons in DOOR, and found 213 operons in common which validate our analysis approach. For example, the operon APP-O279 which contains four genes (*pgaA*, *pgaB*, *pgaC*, *pgaD)* has been characterized as the operon involved in biofilm formation of *A*. *pleuropneumoniae* [78]. Operons encoding known virulence genes of *A*. *pleuropneumoniae* were also identified, such as *cps3ABCD* (APJL1611-1614) encoding CPS biosynthetic enzymes, *cpxDCBA* (APJL1615-1618) encoding proteins involved in the export of CPS and *apxIIICABD* (APJL1344-1347) encoding the ApxIII structure, activation and secretion proteins [79]. Remarkably, we found that the uncharacterized gene APJL0965 was located in *apxII* operon which has been reported to consist of *apxIICA* and a partial *apxIIB’* without apxIID [79]. The product encoded by APJL0965 may be functional as a secretion protein of ApxII. We also identified 34 operons not predicted by the computational operon prediction method. A new operon APP-O25 containing two genes, *putA* and *putP*, annotated as bifunctional proline dehydrogenase/pyrroline-5-carboxylate dehydrogenase and proline symporter/permease was not predicted by DOOR. The functions of putAP gene products are quite conserved among different bacteria [80, 81]. In *E*. *coli* and *S*. *typhimurium*, the orthologs of these genes are well known to form the proline utilization operon [82, 83]. Our results presented the first operon map of *A*. *pleuropneumoniae* and also demonstrated RNA-Seq can improve operon identification in bacterial genomes.

## Conclusion

In summary, a global transcriptome profiling of *A*. *pleuropneumoniae* at single nucleotide resolution using deep RNA-seq was presented in this work. This study confirmed previously annotated open reading frames and provided a more accurate map of gene boundaries. The analysis also identified 32 novel protein-coding genes and 51 sRNAs, defined UTRs regions and generated complete operon structures throughout the genome. The analysis results indicated high complexity of the *A*. *pleuropneumoniae* transcriptome. The functional elements on a whole genome level identified in this study provide a useful reference transcriptome to comprehensively understand the *A*. *pleuropneumoniae* survival and infection process.

## Supporting Information

S1 Fig The FASTQC quality plot of the raw data. (TIF)

S2 Fig Growth curve and RNA isolation of *A*. *pleuropneumoniae* JL03. (A) The time of cell harvest for RNA sequencing is at mid-log phase (optical density at 600 nm = 1.0). (B) Total RNA was extracted from cells and verified for integrity on a 1% agarose gel.
(TIF)

S1 Table Genes with UTR. List of the UTRs and their genome location identified in this study.
(XLS)

S2 Table Comparison of co-expressed gene pairs identified from RNA-Seq data and DOOR program. Line A has a list of the co-expressed gene pairs predicted by DOOR program. Line B has a list of the co-expressed gene pairs predicted by RNA-seq. Line C has a list of the co-expressed gene pairs common to both DOOR and RNA-Seq. Line D has a list of the co-expressed gene pairs unique in RNA-Seq experiment.
(XLS)

S3 Table Comparison of operons predicted from RNA-Seq data and DOOR program. Operons identified by RNA-seq were compared with the operons predicted by DOOR database. The operons along with their ID and genes are listed. Operon type: A- operons common to both DOOR and RNA-Seq; B- operons based on RNA-seq data and not predicted by DOOR; C-operon having different compositions.
(XLS)

## References

[1] Land M, Hauser L, Jun SR, Nookaew I, Leuze MR, Ahn TH, et al Insights from 20 years of bacterial genome sequencing. Functional & integrative genomics. 2015;15(2):141–61. 10.1007/s10142-015-0433-4 25722247 PMC4361730

[2] Sorek R, Cossart P. Prokaryotic transcriptomics: a new view on regulation, physiology and pathogenicity. Nature reviews Genetics. 2010;11(1):9–16. 10.1038/nrg2695 . 19935729

[3] Wang Z, Gerstein M, Snyder M. RNA-Seq: a revolutionary tool for transcriptomics. Nature reviews Genetics. 2009;10(1):57–63. 10.1038/nrg2484 19015660 PMC2949280

[4] Croucher NJ, Thomson NR. Studying bacterial transcriptomes using RNA-seq. Current opinion in microbiology. 2010;13(5):619–24. 10.1016/j.mib.2010.09.009. WOS:000283915900012. 20888288 PMC3025319

[5] Kogenaru S, Qing Y, Guo YP, Wang NA. RNA-seq and microarray complement each other in transcriptome profiling. BMC genomics. 2012;13 Artn 629 10.1186/1471-2164-13-629. WOS:000312961000001. PMC3534599 23153100

[6] Sharma CM, Hoffmann S, Darfeuille F, Reignier J, Findeiss S, Sittka A, et al The primary transcriptome of the major human pathogen Helicobacter pylori. Nature. 2010;464(7286):250–5. 10.1038/nature08756 . 20164839

[7] Tisserant E, Da Silva C, Kohler A, Morin E, Wincker P, Martin F. Deep RNA sequencing improved the structural annotation of the Tuber melanosporum transcriptome. The New phytologist. 2011;189(3):883–91. 10.1111/j.1469-8137.2010.03597.x . 21223284

[8] Wang S, Dong X, Zhu Y, Wang C, Sun G, Luo T, et al Revealing of Mycobacterium marinum transcriptome by RNA-seq. PloS one. 2013;8(9):e75828 10.1371/journal.pone.0075828 24098731 PMC3786904

[9] Perkins TT, Kingsley RA, Fookes MC, Gardner PP, James KD, Yu L, et al A strand-specific RNA-Seq analysis of the transcriptome of the typhoid bacillus Salmonella typhi. PLoS genetics. 2009;5(7):e1000569 10.1371/journal.pgen.1000569 19609351 PMC2704369

[10] Kumar R, Lawrence ML, Watt J, Cooksey AM, Burgess SC, Nanduri B. RNA-seq based transcriptional map of bovine respiratory disease pathogen "Histophilus somni 2336". PLoS One. 2012;7(1):e29435 10.1371/journal.pone.0029435 22276113 PMC3262788

[11] Bosse JT, Janson H, Sheehan BJ, Beddek AJ, Rycroft AN, Kroll JS, et al Actinobacillus pleuropneumoniae: pathobiology and pathogenesis of infection. Microbes Infect. 2002;4(2):225–35. Epub 2002/03/07. S1286457901015349 [pii]. . 11880056 10.1016/s1286-4579(01)01534-9

[12] Xu Z, Zhou Y, Li L, Zhou R, Xiao S, Wan Y, et al Genome biology of Actinobacillus pleuropneumoniae JL03, an isolate of serotype 3 prevalent in China. PloS one. 2008;3(1):e1450 10.1371/journal.pone.0001450 18197260 PMC2175527

[13] Xu Z, Chen X, Li L, Li T, Wang S, Chen H, et al Comparative genomic characterization of Actinobacillus pleuropneumoniae. Journal of bacteriology. 2010;192(21):5625–36. 10.1128/JB.00535-10 20802045 PMC2953695

[14] FASTQC. Available from: http://www.bioinformatics.bbsrc.ac.uk/projects/fastqc/.

[15] Bolger AM, Lohse M, Usadel B. Trimmomatic: a flexible trimmer for Illumina sequence data. Bioinformatics. 2014;30(15):2114–20. 10.1093/bioinformatics/btu170. WOS:000340049100004. 24695404 PMC4103590

[16] Li H, Durbin R. Fast and accurate short read alignment with Burrows-Wheeler transform. Bioinformatics. 2009;25(14):1754–60. 10.1093/bioinformatics/btp324 19451168 PMC2705234

[17] Li H, Ruan J, Durbin R. Mapping short DNA sequencing reads and calling variants using mapping quality scores. Genome research. 2008;18(11):1851–8. 10.1101/gr.078212.108 18714091 PMC2577856

[18] Li H, Handsaker B, Wysoker A, Fennell T, Ruan J, Homer N, et al The Sequence Alignment/Map format and SAMtools. Bioinformatics. 2009;25(16):2078–9. 10.1093/bioinformatics/btp352 19505943 PMC2723002

[19] Carver T, Harris SR, Berriman M, Parkhill J, McQuillan JA. Artemis: an integrated platform for visualization and analysis of high-throughput sequence-based experimental data. Bioinformatics. 2012;28(4):464–9. 10.1093/bioinformatics/btr703 22199388 PMC3278759

[20] Wurtzel O, Sapra R, Chen F, Zhu Y, Simmons BA, Sorek R. A single-base resolution map of an archaeal transcriptome. Genome research. 2010;20(1):133–41. 10.1101/gr.100396.109 19884261 PMC2798825

[21] Mao F, Dam P, Chou J, Olman V, Xu Y. DOOR: a database for prokaryotic operons. Nucleic acids research. 2009;37(Database issue):D459–63. 10.1093/nar/gkn757 18988623 PMC2686520

[22] Pridmore A, Burch D, Lees P. Determination of minimum inhibitory and minimum bactericidal concentrations of tiamulin against field isolates of Actinobacillus pleuropneumoniae. Vet Microbiol. 2011;151(3–4):409–12. Epub 2011/04/19. 10.1016/j.vetmic.2011.03.016 . 21497460

[23] Reese MG. Application of a time-delay neural network to promoter annotation in the Drosophila melanogaster genome. Computers & chemistry. 2001;26(1):51–6. . 11765852 10.1016/s0097-8485(01)00099-7

[24] Kingsford CL, Ayanbule K, Salzberg SL. Rapid, accurate, computational discovery of Rho-independent transcription terminators illuminates their relationship to DNA uptake. Genome biology. 2007;8(2):R22 10.1186/gb-2007-8-2-r22 17313685 PMC1852404

[25] Griffiths-Jones S, Moxon S, Marshall M, Khanna A, Eddy SR, Bateman A. Rfam: annotating non-coding RNAs in complete genomes. Nucleic acids research. 2005;33(Database issue):D121–4. 10.1093/nar/gki081 15608160 PMC540035

[26] Kumar R, Burgess SC, Lawrence ML, Nanduri B. TAAPP: Tiling Array Analysis Pipeline for Prokaryotes. Genomics, Proteomics & Bioinformatics. 2011;9(1–2):56–62. 10.1016/s1672-0229(11)60008-9 PMC5054164 21641563

[27] David L, Huber W, Granovskaia M, Toedling J, Palm CJ, Bofkin L, et al A high-resolution map of transcription in the yeast genome. Proceedings of the National Academy of Sciences of the United States of America. 2006;103(14):5320–5. 10.1073/pnas.0601091103 16569694 PMC1414796

[28] Kaufmann M. The role of the COG database in comparative and functional genomics. Curr Bioinform. 2006;1(3):291–300. 10.2174/157489306777828017. WOS:000244444300003.

[29] Bratlie MS, Johansen J, Drablos F. Relationship between operon preference and functional properties of persistent genes in bacterial genomes. Bmc Genomics. 2010;11 Artn 71 10.1186/1471-2164-11-71. WOS:000275626700001. PMC2837039 20109203

[30] Sabatti C, Rohlin L, Oh MK, Liao JC. Co-expression pattern from DNA microarray experiments as a tool for operon prediction. Nucleic Acids Res. 2002;30(13):2886–93. 10.1093/Nar/Gkf388. WOS:000176607000021. 12087173 PMC117043

[31] Landt SG, Abeliuk E, McGrath PT, Lesley JA, McAdams HH, Shapiro L. Small non-coding RNAs in Caulobacter crescentus. Molecular microbiology. 2008;68(3):600–14. 10.1111/j.1365-2958.2008.06172.x . 18373523 PMC7540941

[32] Gripenland J, Netterling S, Loh E, Tiensuu T, Toledo-Arana A, Johansson J. RNAs: regulators of bacterial virulence. Nature reviews Microbiology. 2010;8(12):857–66. 10.1038/nrmicro2457 . 21079634

[33] Storz G, Vogel J, Wassarman KM. Regulation by small RNAs in bacteria: expanding frontiers. Molecular cell. 2011;43(6):880–91. 10.1016/j.molcel.2011.08.022 21925377 PMC3176440

[34] Repoila F, Darfeuille F. Small regulatory non-coding RNAs in bacteria: physiology and mechanistic aspects. Biology of the Cell. 2009;101(2):117–31. 10.1042/bc20070137 19076068

[35] Saito S, Kakeshita H, Nakamura K. Novel small RNA-encoding genes in the intergenic regions of Bacillus subtilis. Gene. 2009;428(1–2):2–8. 10.1016/j.gene.2008.09.024. WOS:000262071000002. 18948176

[36] Reddy JS, Kumar R, Watt JM, Lawrence ML, Burgess SC, Nanduri B. Transcriptome profile of a bovine respiratory disease pathogen: Mannheimia haemolytica PHL213. BMC bioinformatics. 2012;13 Suppl 15:S4 10.1186/1471-2105-13-S15-S4 23046475 PMC3439734

[37] Liu T, Ren XW, Xiao TF, Yang J, Xu XY, Dong J, et al Identification and characterisation of non-coding small RNAs in the pathogenic filamentous fungus Trichophyton rubrum. BMC genomics. 2013;14 Artn 931 10.1186/1471-2164-14-931. WOS:000329614300001. PMC3890542 24377353

[38] Gomez-Lozano M, Marvig RL, Tulstrup MVL, Molin S. Expression of antisense small RNAs in response to stress in Pseudomonas aeruginosa. BMC genomics. 2014;15 Artn 783 10.1186/1471-2164-15-783. WOS:000342462500001. PMC4180829 25213728

[39] Chao Y, Papenfort K, Reinhardt R, Sharma CM, Vogel J. An atlas of Hfq-bound transcripts reveals 3' UTRs as a genomic reservoir of regulatory small RNAs. The EMBO journal. 2012;31(20):4005–19. 10.1038/emboj.2012.229 22922465 PMC3474919

[40] Oliver HF, Orsi RH, Ponnala L, Keich U, Wang W, Sun Q, et al Deep RNA sequencing of L. monocytogenes reveals overlapping and extensive stationary phase and sigma B-dependent transcriptomes, including multiple highly transcribed noncoding RNAs. BMC genomics. 2009;10:641 10.1186/1471-2164-10-641 20042087 PMC2813243

[41] Gundogdu O, Bentley SD, Holden MT, Parkhill J, Dorrell N, Wren BW. Re-annotation and re-analysis of the Campylobacter jejuni NCTC11168 genome sequence. BMC genomics. 2007;8:162 10.1186/1471-2164-8-162 17565669 PMC1899501

[42] Gao J, Wang J. Re-annotation of two hyperthermophilic archaea Pyrococcus abyssi GE5 and Pyrococcus furiosus DSM 3638. Current microbiology. 2012;64(2):118–29. 10.1007/s00284-011-0035-x . 22057919

[43] Marcellin E, Licona-Cassani C, Mercer TR, Palfreyman RW, Nielsen LK. Re-annotation of the Saccharopolyspora erythraea genome using a systems biology approach. BMC genomics. 2013;14 Artn 699 10.1186/1471-2164-14-699. WOS:000328631200003. PMC4008361 24118942

[44] Guo FB, Xiong L, Teng JL, Yuen KY, Lau SK, Woo PC. Re-annotation of protein-coding genes in 10 complete genomes of Neisseriaceae family by combining similarity-based and composition-based methods. DNA research: an international journal for rapid publication of reports on genes and genomes. 2013;20(3):273–86. 10.1093/dnares/dst009 23571676 PMC3686433

[45] Biao Guo F, Lin Y, Ling Chen L. Recognition of Protein-coding Genes Based on Z-curve Algorithms. Current genomics. 2014;15(2):95–103. 10.2174/1389202915999140328162724 24822027 PMC4009845

[46] den Bakker HC, Cummings CA, Ferreira V, Vatta P, Orsi RH, Degoricija L, et al Comparative genomics of the bacterial genus Listeria: Genome evolution is characterized by limited gene acquisition and limited gene loss. BMC genomics. 2010;11:688 10.1186/1471-2164-11-688 21126366 PMC3019230

[47] Gao XY, Zhi XY, Li HW, Klenk HP, Li WJ. Comparative genomics of the bacterial genus Streptococcus illuminates evolutionary implications of species groups. PloS one. 2014;9(6):e101229 10.1371/journal.pone.0101229 24977706 PMC4076318

[48] Tjaden B, Saxena RM, Stolyar S, Haynor DR, Kolker E, Rosenow C. Transcriptome analysis of Escherichia coli using high-density oligonucleotide probe arrays. Nucleic Acids Res. 2002;30(17):3732–8. 10.1093/Nar/Gkf505. WOS:000177998100015. 12202758 PMC137427

[49] Akama T, Suzuki K, Tanigawa K, Kawashima A, Wu H, Nakata N, et al Whole-genome tiling array analysis of Mycobacterium leprae RNA reveals high expression of pseudogenes and noncoding regions. Journal of bacteriology. 2009;191(10):3321–7. 10.1128/JB.00120-09 19286800 PMC2687151

[50] Ignatov D, Malakho S, Majorov K, Skvortsov T, Apt A, Azhikina T. RNA-Seq Analysis of Mycobacterium avium Non-Coding Transcriptome. PloS one. 2013;8(9). ARTN e74209 10.1371/journal.pone.0074209. WOS:000324494000104. PMC3774663 24066122

[51] Osmundson J, Dewell S, Darst SA. RNA-Seq reveals differential gene expression in Staphylococcus aureus with single-nucleotide resolution. PloS one. 2013;8(10):e76572 10.1371/journal.pone.0076572 24116120 PMC3792026

[52] Wurtzel O, Yoder-Himes DR, Han K, Dandekar AA, Edelheit S, Greenberg EP, et al The single-nucleotide resolution transcriptome of Pseudomonas aeruginosa grown in body temperature. PLoS pathogens. 2012;8(9):e1002945 10.1371/journal.ppat.1002945 23028334 PMC3460626

[53] Wang Y, Li X, Mao Y, Blaschek HP. Single-nucleotide resolution analysis of the transcriptome structure of Clostridium beijerinckii NCIMB 8052 using RNA-Seq. BMC genomics. 2011;12:479 10.1186/1471-2164-12-479 21962126 PMC3271303

[54] Pfeifer-Sancar K, Mentz A, Ruckert C, Kalinowski J. Comprehensive analysis of the Corynebacterium glutamicum transcriptome using an improved RNAseq technique. BMC genomics. 2013;14 Artn 888 10.1186/1471-2164-14-888. WOS:000329612300001. PMC3890552 24341750

[55] Merino S, Knirel YA, Regue M, Tomas JM. Experimental Identification of Actinobacillus pleuropneumoniae Strains L20 and JL03 Heptosyltransferases, Evidence for a New Heptosyltransferase Signature Sequence. PloS one. 2013;8(1). ARTN e55546 10.1371/journal.pone.0055546. WOS:000315563800164. PMC3559599 23383222

[56] Li L, Zhu J, Yang K, Xu Z, Liu Z, Zhou R. Changes in gene expression of Actinobacillus pleuropneumoniae in response to anaerobic stress reveal induction of central metabolism and biofilm formation. Journal of microbiology. 2014;52(6):473–81. 10.1007/s12275-014-3456-y . 24723105

[57] Deslandes V, Denicourt M, Girard C, Harel J, Nash JH, Jacques M. Transcriptional profiling of Actinobacillus pleuropneumoniae during the acute phase of a natural infection in pigs. BMC Genomics. 2010;11:98 Epub 2010/02/10. 10.1186/1471-2164-11-98 20141640 PMC2829017

[58] Klitgaard K, Friis C, Angen O, Boye M. Comparative profiling of the transcriptional response to iron restriction in six serotypes of Actinobacillus pleuropneumoniae with different virulence potential. BMC Genomics. 2010;11:698 Epub 2010/12/15. 10.1186/1471-2164-11-698 21143895 PMC3091793

[59] Yoder-Himes DR, Chain PS, Zhu Y, Wurtzel O, Rubin EM, Tiedje JM, et al Mapping the Burkholderia cenocepacia niche response via high-throughput sequencing. Proceedings of the National Academy of Sciences of the United States of America. 2009;106(10):3976–81. 10.1073/pnas.0813403106 19234113 PMC2645912

[60] Zeng Q, Sundin GW. Genome-wide identification of Hfq-regulated small RNAs in the fire blight pathogen Erwinia amylovora discovered virulence-regulating small RNAs. Phytopathology. 2014;104(11):134–5. WOS:000346303300769. 24885615 10.1186/1471-2164-15-414 PMC4070566

[61] Trotochaud AE, Wassarman KM. 6S RNA function enhances long-term cell survival. Journal of bacteriology. 2004;186(15):4978–85. 10.1128/JB.186.15.4978-4985.2004 15262935 PMC451630

[62] Cavanagh AT, Wassarman KM. 6S RNA, a global regulator of transcription in Escherichia coli, Bacillus subtilis, and beyond. Annual review of microbiology. 2014;68:45–60. 10.1146/annurev-micro-092611-150135 . 24742053

[63] Urbanowski ML, Stauffer LT, Stauffer GV. The gcvB gene encodes a small untranslated RNA involved in expression of the dipeptide and oligopeptide transport systems in Escherichia coli. Molecular microbiology. 2000;37(4):856–68. . 10972807 10.1046/j.1365-2958.2000.02051.x

[64] McArthur SD, Pulvermacher SC, Stauffer GV. The Yersinia pestis gcvB gene encodes two small regulatory RNA molecules. BMC microbiology. 2006;6:52 10.1186/1471-2180-6-52 16768793 PMC1557403

[65] Vogel J. A rough guide to the non-coding RNA world of Salmonella. Molecular microbiology. 2009;71(1):1–11. 10.1111/j.1365-2958.2008.06505.x . 19007416

[66] Pulvermacher SC, Stauffer LT, Stauffer GV. Role of the Escherichia coli Hfq protein in GcvB regulation of oppA and dppA mRNAs. Microbiology. 2009;155(Pt 1):115–23. 10.1099/mic.0.023432-0 . 19118352

[67] Livny J, Waldor MK. Identification of small RNAs in diverse bacterial species. Current opinion in microbiology. 2007;10(2):96–101. 10.1016/j.mib.2007.03.005 . 17383222

[68] Papenfort K, Vogel J. Regulatory RNA in bacterial pathogens. Cell host & microbe. 2010;8(1):116–27. 10.1016/j.chom.2010.06.008 . 20638647

[69] McClure R, Balasubramanian D, Sun Y, Bobrovskyy M, Sumby P, Genco CA, et al Computational analysis of bacterial RNA-Seq data. Nucleic acids research. 2013;41(14):e140 10.1093/nar/gkt444 23716638 PMC3737546

[70] Regulski EE, Moy RH, Weinberg Z, Barrick JE, Yao Z, Ruzzo WL, et al A widespread riboswitch candidate that controls bacterial genes involved in molybdenum cofactor and tungsten cofactor metabolism. Molecular microbiology. 2008;68(4):918–32. 10.1111/j.1365-2958.2008.06208.x 18363797 PMC2408646

[71] Horn G, Hofweber R, Kremer W, Kalbitzer HR. Structure and function of bacterial cold shock proteins. Cell Mol Life Sci. 2007;64(12):1457–70. 10.1007/s00018-007-6388-4. WOS:000247272100003. 17437059 PMC11138454

[72] Domka J, Lee J, Wood TK. YliH (BssR) and YceP (BssS) regulate Escherichia coli K-12 biofilm formation by influencing cell signaling. Appl Environ Microb. 2006;72(4):2449–59. 10.1128/Aem.72.4.2449-2459.2006. WOS:000236749400022. PMC1448992 16597943

[73] Deslandes V, Denicourt M, Girard C, Harel J, Nash JHE, Jacques M. Transcriptional profiling of Actinobacillus pleuropneumoniae during the acute phase of a natural infection in pigs. BMC genomics. 2010;11 Artn 98 10.1186/1471-2164-11-98. WOS:000275292200002. PMC2829017 20141640

[74] Li L, Xu ZF, Zhou Y, Sun LL, Liu ZD, Chen HC, et al Global Effects of Catecholamines on Actinobacillus pleuropneumoniae Gene Expression. PloS one. 2012;7(2). ARTN e31121 10.1371/journal.pone.0031121. WOS:000302730100043. PMC3275570 22347439

[75] Giuliodori AM, Di Pietro F, Marzi S, Masquida B, Wagner R, Romby P, et al The cspA mRNA is a thermosensor that modulates translation of the cold-shock protein CspA. Molecular cell. 2010;37(1):21–33. 10.1016/j.molcel.2009.11.033 . 20129052

[76] Livny J, Waldor MK. Mining regulatory 5'UTRs from cDNA deep sequencing datasets. Nucleic acids research. 2010;38(5):1504–14. 10.1093/nar/gkp1121 19969537 PMC2836559

[77] Chan CL, Landick R. The Salmonella typhimurium his operon leader region contains an RNA hairpin-dependent transcription pause site. Mechanistic implications of the effect on pausing of altered RNA hairpins. The Journal of biological chemistry. 1989;264(34):20796–804. . 2479649

[78] Bosse JT, Sinha S, Li MS, O'Dwyer CA, Nash JH, Rycroft AN, et al Regulation of pga operon expression and biofilm formation in Actinobacillus pleuropneumoniae by sigmaE and H-NS. Journal of bacteriology. 2010;192(9):2414–23. 10.1128/JB.01513-09 20207760 PMC2863473

[79] Frey J. Virulence in Actinobacillus pleuropneumoniae and RTX toxins. Trends Microbiol. 1995;3(7):257–61. Epub 1995/07/01. S0966-842X(00)88939-8 [pii]. . 7551637 10.1016/s0966-842x(00)88939-8

[80] Cho K, Winans SC. The putA gene of Agrobacterium tumefaciens is transcriptionally activated in response to proline by an Lrp-like protein and is not autoregulated. Molecular microbiology. 1996;22(5):1025–33. . 8971722 10.1046/j.1365-2958.1996.01524.x

[81] Lee JH, Park NY, Lee MH, Choi SH. Characterization of the Vibrio vulnificus putAP Operon, Encoding Proline Dehydrogenase and Proline Permease, and Its Differential Expression in Response to Osmotic Stress. Journal of bacteriology. 2003;185(13):3842–52. 10.1128/jb.185.13.3842-3852.2003 12813078 PMC161561

[82] Zhang M, White TA, Schuermann JP, Baban BA, Becker DF, Tanner JJ. Structures of the Escherichia coli PutA proline dehydrogenase domain in complex with competitive inhibitors. Biochemistry. 2004;43(39):12539–48. 10.1021/bi048737e 15449943 PMC3727243

[83] Muro-Pastor AM, Maloy S. Proline dehydrogenase activity of the transcriptional repressor PutA is required for induction of the put operon by proline. The Journal of biological chemistry. 1995;270(17):9819–27. . 7730362 10.1074/jbc.270.17.9819

