# Supplementary figures and images for: A Transcriptome Map of Actinobacillus pleuropneumoniae at Single-Nucleotide Resolution Using Deep RNA-Seq
Source: PLoS One. 2016 Mar 28;11(3):e0152363. doi: 10.1371/journal.pone.0152363 (PMC4809551; doi:10.1371/journal.pone.0152363)

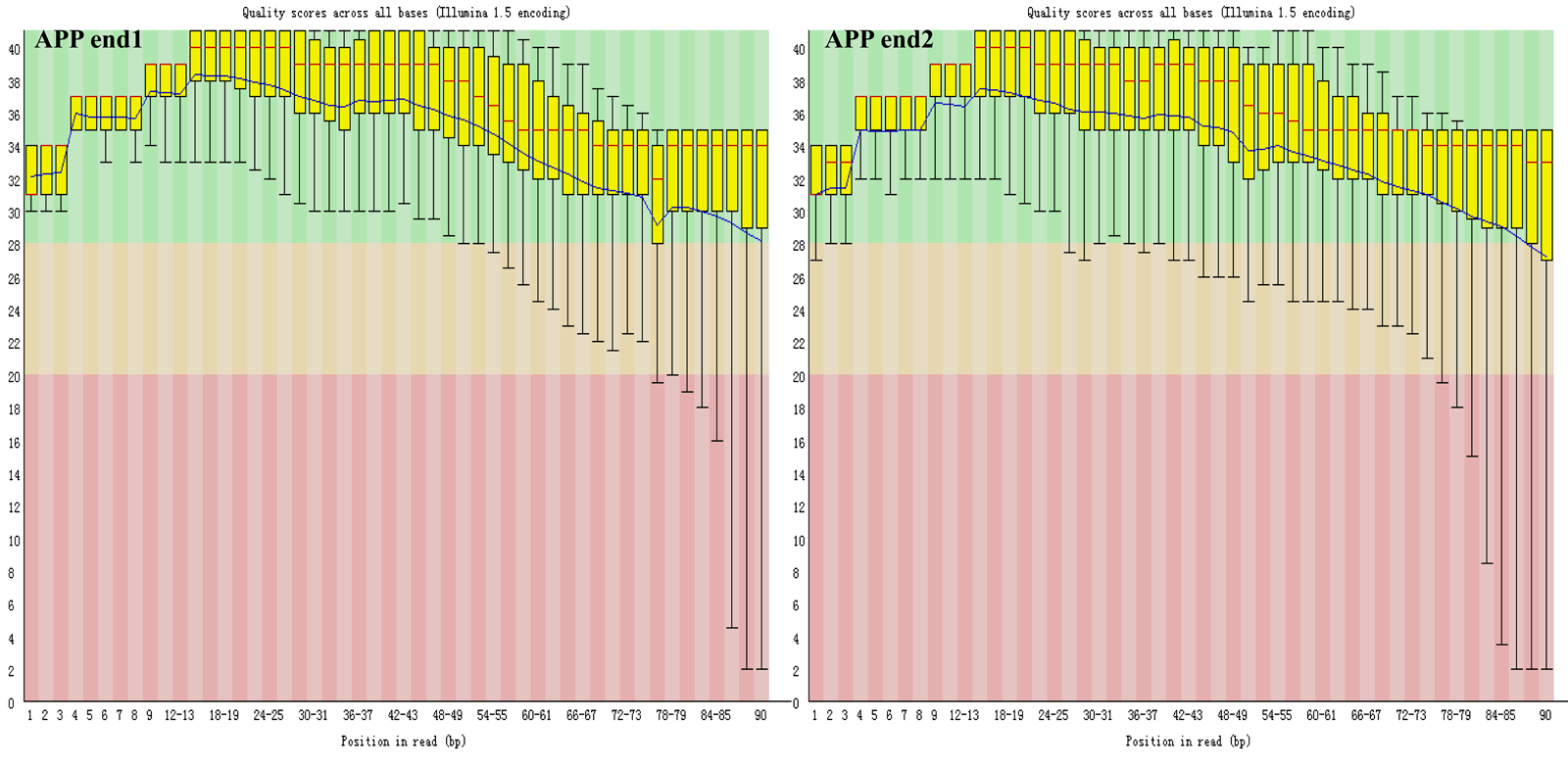

Supplement: S1 Fig — (TIF) [file pone.0152363.s001.tif]

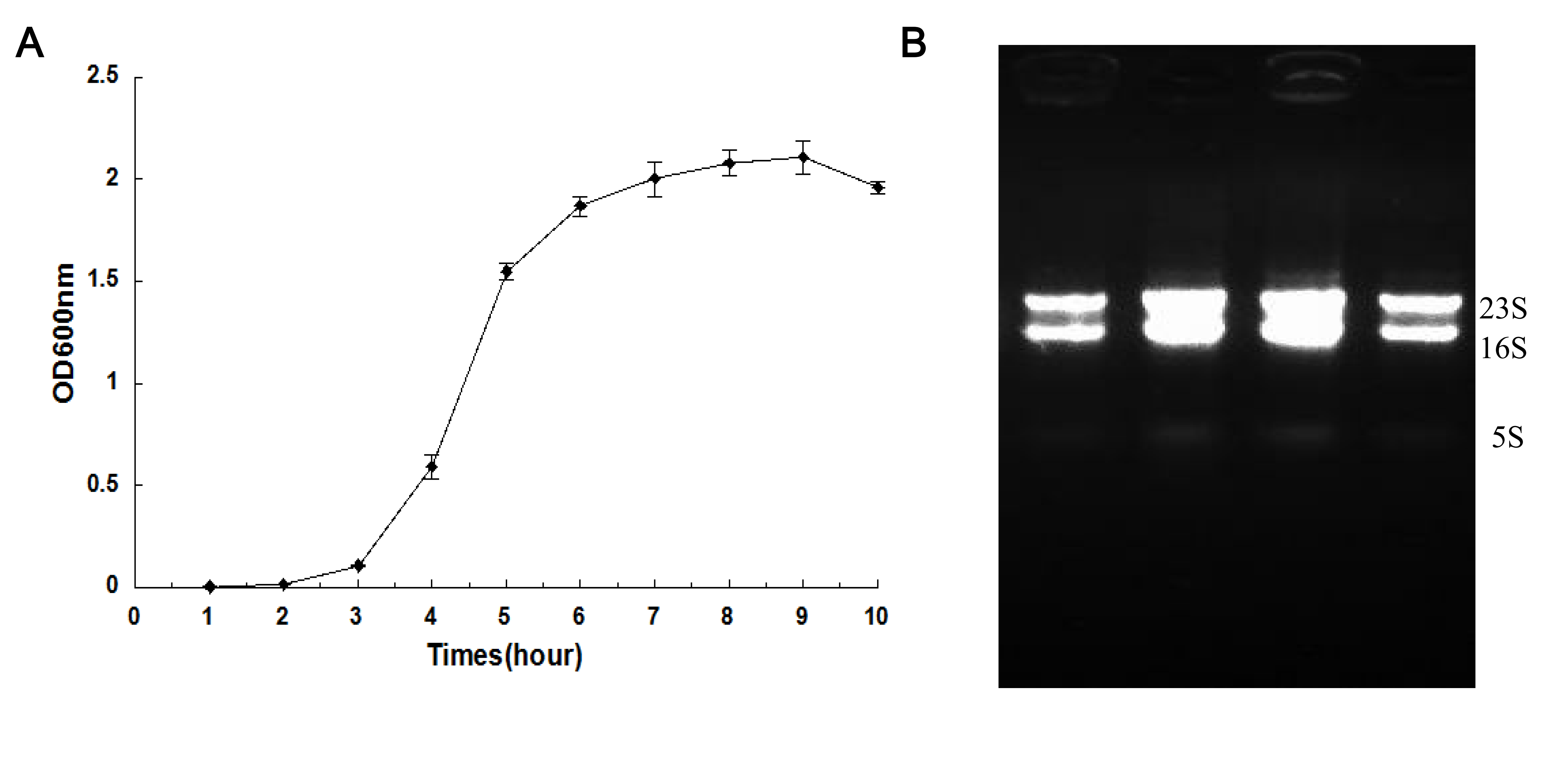

Supplement: S2 Fig — (A) The time of cell harvest for RNA sequencing is at mid-log phase (optical density at 600 nm = 1.0). (B) Total RNA was extracted from cells and verified for integrity on a 1% agarose gel. (TIF) [file pone.0152363.s002.tif]
